# Social cognition in individuals with 22q11.2 deletion syndrome and its link with psychopathology and social outcomes: a review

**DOI:** 10.1186/s12888-020-02975-5

**Published:** 2021-03-06

**Authors:** Branka Milic, Clémence Feller, Maude Schneider, Martin Debbané, Henriette Loeffler-Stastka

**Affiliations:** 1 Clinic for Psychoanalysis and Psychotherapy, Medical University of Vienna, Vienna, Austria; 2 Clinical Psychology Unit for Intellectual and Developmental Disabilities, Faculty of Psychology and Educational Sciences, University of Geneva, Geneva, Switzerland; 3 Developmental Clinical Psychology Unit, Faculty of Psychology and Educational Sciences, University of Geneva, Geneva, Switzerland; 4 Research Department of Clinical, Educational and Health Psychology, University College London, London, UK

**Keywords:** 22q11DS, Social cognition, Emotion processing, Theory of mind, ToM, Mentalizing, Psychopathology, Schizophrenia, Psychosis, Social outcomes, Preventive interventions

## Abstract

**Background:**

The 22q11.2 deletion syndrome (22q11DS) is a genetic syndrome that results in a highly variable profile of affected individuals of which impairments in the social domain and increased psychopathology are the most prominent. Notably, 25–30% of affected individuals eventually develop schizophrenia/psychosis, predisposing persons with the syndrome to increased risk for this disorder. Because social cognition is considered to underlie social behavior and to be related to psychopathology, this systematic review investigated social cognition in individuals with 22q11DS and examined reported links across its domains with psychopathology and social outcomes. This can provide the basis for a closer understanding of the path from risk to disorder and will inform on the specific domains that can be targeted with preventive intervention strategies.

**Method:**

Systematic literature review of studies that reported the links between social cognitive domains and psychopathology and/or social outcomes in individuals with 22q11DS. Electronic databases searched were PubMed and PsycINFO.

**Results:**

Defined eligibility criteria identified a total of ten studies to be included in the present review. Selected studies investigated links between two domains of social cognition (emotion processing and theory of mind (ToM)) and psychopathology and/or social outcomes. With respect to the links to psychopathology, two aspects of social cognition were related primarily to negative symptoms. Results regarding the associations to positive and emotional symptoms (anxiety/depression) are limited and require further investigation. Even though both aspects of social cognition were associated with social outcomes, several studies also found no links between these two domains. Both reports invite for an additional examination of reported results and specific considerations regarding chosen constructs.

**Conclusion:**

Although equivocal, results of the present review provide sufficient evidence that social cognition is a useful domain for the closer elucidation of clinical outcomes and social difficulties in this population. At the same time, longitudinal studies and consideration of other variables are also necessary for a timely understanding of affected persons in this respect.

**Supplementary Information:**

The online version contains supplementary material available at 10.1186/s12888-020-02975-5.

## Background

The 22q11.2 deletion syndrome (22q11DS) is a genetic syndrome associated with a microdeletion on the long arm of chromosome 22, which is appointed as 22q11.2 deletion [1]. Commonly, 22q11 deletion comprises about 50 genes, with a reported prevalence of 1 in every 2000 [2], 4000 [2, 3] to 6000 [4] births, and in over 90% of the cases is de novo [5]. Given that 25–30% of affected individuals eventually develop schizophrenia/psychosis, 22q11DS is acknowledged as the second-highest genetic risk factor for the development of schizophrenia. Additionally, since the phenotypic expression of schizophrenia in 22q11DS is indistinguishable from idiopathic schizophrenia, a closer understanding of affected individuals provides an opportunity for elucidating the trajectory path from risk to disorder [6]. Equally, this can serve as a basis for preventive and intervention strategies designed to ameliorate behavioral and functional challenges encountered by affected persons and their environment.

Even though 22q11DS is known for its inter- and intra-individual phenotypic variability [7], domains that are regularly affected involve cognitive, psychiatric, and social domain. Individuals with 22q11DS often face impairments in neurocognition (working memory, executive function, borderline (IQ, 70–84) to mild (IQ < 70) intellectual disability) and social cognition [8]. Interestingly, cognitive abilities (verbal and performance IQ) are found to be inversely associated with age in this population and more severe intellectual disability is encountered mainly in adults [9–11]. Concerning psychiatric characteristics, behavioral issues are the most common reported symptoms [11] and are often classified under general or emotional symptoms (e.g., depression, anxiety, tension, poor impulse control), negative symptoms (e.g., social anhedonia or withdrawal, avolition, difficulty in abstract thinking), and positive symptoms (e.g., unusual perceptual experiences, delusions, hallucinations) (e.g., [12, 13]). Therefore, diagnostic categories frequently associated with the syndrome involve attention deficit hyperactivity disorder (ADHD), autism spectrum disorders (ASD), anxiety, mood disorders (major depression), and psychosis. Notably, the prevalence of the specific psychiatric categories is also related to the age of affected individuals [8]. Thus, certain diagnostic categories are most frequently encountered in children and adolescents (ADHD, ASD), anxiety can be found in all age groups, and the prevalence of major depression and schizophrenia/psychosis significantly increases with the age of affected individuals [8].

Reports on the social domain are divided. Many studies suggest this domain to be affected, since children with the syndrome are described as being socially immature, withdrawn, shy, and as facing challenges in initiating and forming lasting social relationships (e.g., [14, 15]). By contrast, early studies with very young children confirm no such findings [16, 17]. Nevertheless, the developmental trajectory of behavior in affected individuals tends to shift from an externalizing profile (attention deficit, oppositional, impulsive) encountered at a younger age to an internalizing profile (withdrawn, shy, lack of initiative) that is characteristic for individuals with 22q11DS in adolescence and adulthood [11].

### Social cognition, psychopathology and social outcomes in individuals with 22q11DS

A large number of studies on individuals across the psychosis spectrum (e.g., patients with schizophrenia [18], young people at clinical high risk for psychosis [19], and the population with 22q11DS (e.g., [20, 21])) have reported impairments in the social area. Given that social cognition is considered to underlie social behavior [22] and can serve as a potential marker for schizophrenia and other clinical conditions [23], this specific domain has received considerable attention in research on schizophrenia and 22q11DS.

According to The Social Cognition Psychometric Evaluation (SCOPE) study, there are four major domains of social cognition: emotion processing, theory of mind (ToM), social perception, and attributional style/bias [24]. Emotion processing, also referred to as the “social-perceptual” (affective) aspect of social cognition, refers to the way a person perceives, attends to, and recognizes other people with the ability of a person to correctly detect facial emotions [22]. This domain of social cognition has been extensively researched in 22q11DS and impairments in face memory, face recognition, and emotion identification have been reported in individuals with the syndrome (e.g., [25–29]). Theory of mind (mental state attribution), also referred to as “social-cognitive” (reflective) aspect of social cognition, denotes the ability of an individual to understand that other people have mental states (thoughts, beliefs, intentions) independent and different from one’s own and to make attributions about the mental states of others [30]. This includes understanding false-beliefs, hints, intentions, deception, irony, metaphor, and faux pas [18]. Although studies tend to report deficits in ToM in individuals with 22q11DS (e.g., [21, 26]), worthy of note is the impact of age, since in typical development, this domain continues to improve during adolescence and early adulthood [31]. Social perception (or social knowledge) refers to the individual’s ability to decode and interpret social roles, social rules, and contexts from non-verbal cues (i.e., body language, voice intonation) [32]. Attributional style/bias depicts how persons tend to explain the causes of social interactions and events [33]. To the best of our knowledge, social perception and attributional style/bias have received little or no attention in the field of 22q11DS at this stage. It is significant to note that even though four identified domains of social cognition are described as distinct processes, they are not completely separate. Rather, these constructs interact and require the integration of complex skills for a person to attain socially adaptive behavior [34]. In this respect, social outcomes are often operationalized through assessments of social skills, competence, and/or functioning. Specifically, social skills or competencies denote the behaviors necessary for a person to be engaged in successful interpersonal interactions; social functioning is associated with everyday domains, such as independent living, employment, interpersonal relationships [35, 36].

Interconnection of previously defined concepts and their link with psychopathology has been a topic of extensive research and discussion in the field. By way of example, social cognitive deficits have been consistently confirmed in individuals with schizophrenia and psychosis in all phases of illness [19, 37, 38]. Specific associations have been reported between social cognition and negative symptoms of psychosis in persons with schizophrenia [18]. Similarly, Chow et al. found that individuals with 22q11DS who developed psychosis displayed more severe ToM impairments compared to those who did not develop psychosis [20]. However, it should be noted that deficits in social cognition have been reported in 22q11DS regardless of psychopathology status [39] and even in the absence of a psychotic disorder [40].

Concerning the association between social cognition and social outcomes, findings seem to be divided. Indeed, a meta-analysis on individuals with schizophrenia reported medium to large associations between social cognition and functioning [41] that also proved to be stable over time [32, 42, 43]. Specifically, a deficit in emotion perception and social-cognitive problem-solving in persons with schizophrenia were associated with social competence. Similarly, emotion processing had a consistent relationship with social functioning [44, 45] and ToM was predictive of social skills in individuals with schizophrenia [46]. At the same time, however, several studies reported no significant association between a combined measure of social cognition (emotion processing and ToM) [47] or specifically between ToM [48] and social outcomes in persons with psychosis. In a similar manner, research on these specific associations in persons with 22q11DS has also shown inconsistent results. For instance, Vangkilde at al. examined both aspects of social cognition and found no significant association with social competence, skills, and functioning [49]. In contrast, Campbell et al. reported associations between both domains of social cognition and social outcomes [50].

Even though deficits in social cognition could partly account for social dysfunction and are often related to psychopathology in individuals with 22q11DS, reported evidence that links specific social cognitive domains to clinical and social outcomes has been ambiguous and often contradictory. Therefore, the present paper aims to gather and systematically review studies conducted on individuals with 22q11DS that have examined and reported associations between any domain of social cognition and psychopathology and/or social outcomes. In order to achieve this goal, we will first: systematically select studies on individuals with 22q11DS and social cognition. Second, we will look for the studies that have also assessed psychopathology and social outcomes in affected persons. Third, we will extract the data that reported associations between social cognition and psychopathology and/or social outcomes. Fourth, we will provide a narrative synthesis and interpretation of the reported information and will aim to highlight critical aspects of the current state of research on the topic including plausible clinical implications. This could serve as the basis for future research in the field that can lead to informed decisions on early prevention and management of behavioral and clinical difficulties in 22q11DS and will possibly benefit clinicians involved in their work with this population.

## Method

### Search strategy

Following the Preferred Reporting Items for Systematic Reviews and Metanalysis (PRISMA) [51], a systematic search was conducted using two electronic databases PubMed and PsycINFO until April 29th, 2020. The search terms for 22q11 deletion syndrome (DS) were based on keywords and phrases encountered in the literature related to the syndrome including early publications. The following search terms for 22q11DS involved: “22q11 deletion syndrome,” “22q11,” “velocardiofacial syndrome,” “DiGeorge Syndrome,” “CATCH-22,” “Shprintzen syndrome,” and were combined with OR. Social cognitive domains are defined by the SCOPE study [24] and include: emotion processing, social perception, theory of mind, and attributional style/bias; thus, search terms for this domain were: “social cognition,” “theory of mind,” “social perception,” “facial identification,” “face recognition,” “face memory,” “emotion perception,” “affect recognition,” “emotion processing,” “attributional bias,” and were combined by OR. The final result for the search string regarding 22q11 DS was combined with the final result for the search string regarding social cognition with AND.

Domains related to psychopathology and social outcomes were not part of the search strategy, as the aim was to gather all publications on social cognition in 22q11DS and to manually select studies that fitted eligibility criteria. Reference lists and studies from other sources were also searched for possible relevant articles.

### Eligibility criteria

Papers were eligible to be included in the review if they met the following inclusion criteria: 1) research involving participants with a confirmed 22q11.2 deletion syndrome, 2) at least one task assessing social cognition, 3) at least one assessment of psychopathology and social outcomes, 4) reported associations between social cognition in individuals with 22q11DS and psychopathology and/or social outcomes. Papers were excluded if they: did not meet these criteria, were case studies, were studies reporting results of interventions, and if they were not empirical studies (e.g., review papers, expert opinions, published theses).

### Study selection

**Fig. 1:**
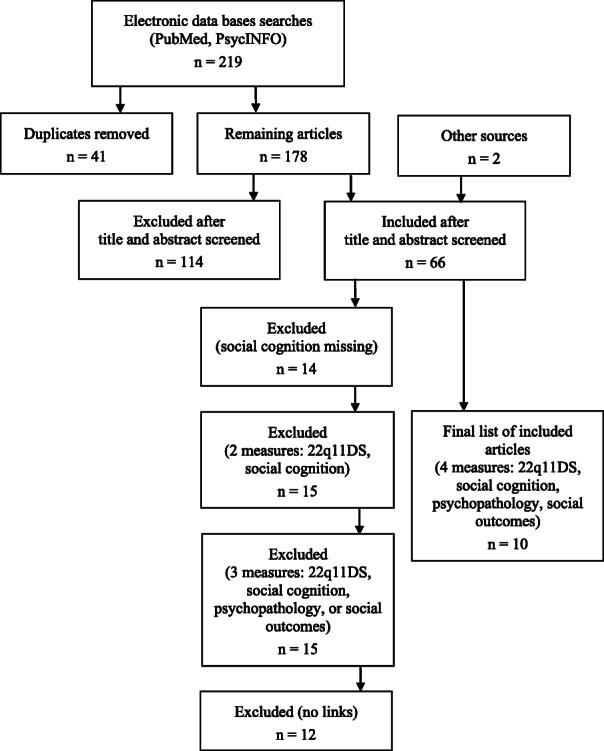
Methodological Flowchart

The first author conducted the search in electronic databases PubMed and PsycINFO. A selection of the studies was then independently done by two authors (BM, CF), thereafter compared, and an agreement for the final inclusion of the studies was reached. The search strategy resulted in a total of 219 retrieved studies. After 41 duplicates were removed, 178 studies were screened based on the titles and abstracts; two studies were added from other sources. In total, sixty-six articles were screened for eligibility criteria based on the full-texts. Twenty-two studies included all four domains of interest. Twelve studies were then excluded, as they have not investigated nor reported associations between social cognition and either psychopathology or social outcomes. The selection process is presented in the methodological flowchart (Fig. 1).

## Results

### Study characteristics

Defined eligibility criteria identified a total of ten studies to be included in the present review (Table 1). The age of participants in the selected studies was between 6 and 33 years old. Five papers involved sample sizes with 24 to 35 participants [49, 54–57]; five articles had more than 40 participants [50, 52, 53, 58, 59]. Two articles had longitudinal designs [58, 59] and one study was conducted at two sites (UCLA, SUNY) [52]. A complete overview of included studies, with main characteristics and assessments for each domain is presented in Table 1.

**Table 1:** Studies that report the links between social cognitive domains and psychopathology and/or social outcomes in individuals with 22q11DS

Author (year)	Country	N	Age M (SD), (range)	Gender N (%F)	IQ (SD), (range)	Detection method	Social Cognition Assessment	Psychopathology Assessment	Social Outcomes Assessment
Campbell et al. (2011) [50]	UK	50	10.99 (2.9), (6–16.75)	28 (56%)	65.8 (9.32), (40–94)	FISH	EP: MRC Face Processing Skills Battery ToM: False-belief stories	SDQ (emotional problems)	SDQ (peer relationship problems)
Ho et al. (2012) [52]	USA	63	13.7 (5.5), (6–25) /17.1 (1.9), (14–22)	11 (38%) /20 (59%)	80.5 (13.7), (−)/ 74.5 (13.2),(−)	Diagnostically confirmed^a^	ToM: The Animations Task	ADOS, ADI-R, SCID	SRS
Shashi et al. (2012) [53]	USA	66	10.5 (2.6), (−)	32 (48%)	(−)	Diagnostically confirmed^a^	EP: DANVA, Child Facial Expressions and the Child Paralanguage Subtests	CBCL, C-DISC, GAF	SSRS
Campbell et al. (2015) [54]	Australia	24	16.75 (−), (12–21)	14 (58%)	75.88 (14.93), (56–115)	Diagnostically confirmed^a^	EP: EAT ToM: PST (false-belief vs. social-script, mechanical, capture)	SCID, K-SADS-PL, SDQ (emotional problems)	SDQ (peer relationship problems)
Vangkilde et al. (2016) [49]	Denmark	29	15.7 (2.8), (−)	19 (65%)	79.52 (−), (35–113)	SNP array analysis	EP: ERT ToM: TASIT	SIPS	ABAS-II, SRS
Badoud et al. (2017) [55]	Switzerland	29	17.79 (2.89), (11–21)	13 (45%)	75.33 (11.66), (−)	QF-PCR	EP: Pictures of Facial Affect ToM: The Director Task	SPQ	VABS
Schneider et al. (2017) [56]	Switzerland	35	18.06 (3.40), (11–24)	21 (60%)	(−)	QF-PCR	EP: BFRT	SPQ,YSR/ASR (anxiety and depression)	DAS (dysfunctional beliefs: negative performance beliefs, need for approval)
Dubourg et al. (2018) [57]	Switzerland	22	20.3 (5.2), (12–32)	17 (77%)	74 (12), (−)	QF-PCR	EP: BFRT Social Perception: IAPS and scrambled images	K-SADS-PL, SCID-I, SIPS, DICA	SRS
Zaharia et al. (2018) [58]	Switzerland	75	Longit. (T1)12.81 (3.57), (−)	40 (53%)	71.96 (11.25), (−)	QF-PCR	EP: Face Discrimination Task (Jane task/featural and configural)	CBCL, ABCL, PANSS	PANSS (poor social abilities - negative symptoms subscale)
Weinberger et al. (2018) [59]	Israel	44	20.7 (6.0), (12–33)	21 (48%)	77.2 (11.0), (−)	FISH	EP: Penn Computerized Neurocognitive Battery (Penn Emotion Identification Test Penn Emotion Differentiation Test Penn Age Differentiation Test)	SIPS/SOPS, K-SADS or SCID	VABS

*ABAS-II* The Adaptive Behavior Assessment System—Sec. Ed., *ABCL* Adult behavior checklist, *ADI-R* Autism Diagnostic Interview-Revised, *ADOS* Autism Diagnostic Observation Schedule, *ASR* adult self-report, *BFRT* Benton Facial Recognition Test, *CBCL* Child behavioral checklist, *C-DISC* Diagnostic Interview Schedule for Children, *DANVA* Diagnostic Analysis of Non-Verbal Accuracy, *DAS* Dysfunctional Attitude Scale-Form A, *DICA* Diagnostic Interview for Children and Adolescents, *EAT* Emotion Attribution Task, *EP* Emotion processing, *ERT* Emotion Recognition Task, *FISH* Fluorescence in Situ Hybridization, *GAF* Global Assess. of Function, *IAPS* International Affective Picture System, *K-SADS-PL* Schedule for Affective Disorders and Schizophrenia for School Age Children - Present and Lifetime, *PANSS* Positive and Negative Syndrome Scale, *PST* Picture Sequencing Task, *QF-PCR* Quantitative Fluorescence Polymerase Chain Reaction, *SADS* Schedule for Affective Disorders and Schizophrenia for School-Age Children, *SCID* Structured Clinical Interview for DSM IV, Axis I Disorders, *SDQ* Strengths and Difficulties, Questionnaire, *SIPS* Structured Interview for Prodr.Syndromes, *SOPS* Scale of Prodromal Symptoms, *SPQ* Schizotypal Personality Questionnaire, *SRS* Social Responsiveness Scale, *SSRS* Social Skills Rating System, *TASIT* The Awareness of Social Inference Test, *ToM* Theory of Mind, *VABS* Vineland Adaptive Behavior Scales, *YSR* youth self-report, ^a^cardiology, paediatrics and/or genetics clinics

Included articles addressed three previously defined domains of social cognition: emotion processing (social-perceptual part), ToM (social-cognitive part), and social perception; no study investigated attributional style/bias. Specifically, nine studies assessed emotion processing (social-perceptual aspect) [49, 50, 53–59], five papers examined social-cognitive part (ToM) [49, 50, 52, 54, 55], and one article investigated social perception [57]; four studies involved assessments of (both) social-perceptual and social-cognitive aspects of social cognition [49, 50, 54, 55]. Descriptions of the tasks used to assess social cognition (emotion processing, ToM, and social perception) in the included papers are presented in the Additional file 1.

All nine studies that assessed emotion processing (social-perceptual part) reported deficits in individuals with 22q11DS. The only study that found no significant difference between individuals with 22q11DS and the control group in emotion recognition used a different form of assessment (by the tone of voice) [53], compared to the remaining studies that examined the recognition of facial expressions.

Findings on the assessment of ToM (social-cognitive part) were not as consistent. Although all five studies that evaluated this aspect of social cognition reported deficits in individuals with 22q11DS [49, 50, 52, 54, 55], some acknowledged no difference with the control group in response times [54, 55] or for simpler (first-order) stories/tasks [50].

The only study that assessed social perception in individuals with 22q11DS [57] found reduced brain activation in regions belonging to the default mode network (DMN) (except for temporal lobes) during the perception of social information compared to controls. However, the between-group comparison of social perception did not reveal any significant difference between the two groups. The same result was found for the influence of emotions on social perception (in within- and between-group comparisons) [57].

Concerning psychopathology and social outcomes, all included studies reported elevated symptoms (general, negative, positive) and poorer social outcomes in individuals with 22q11DS. One article reported similar results between the groups on cognitive-perceptual (unusual perceptual experiences, paranoid ideation) and disorganised dimension (odd behavior and speech) of schizotypy [55]; additionally, one paper found no differences in prosocial behavior [54]. Assessments of psychopathology and social outcomes are listed in Table 1 and Additional file 1.

### Links between social cognitive domains and psychopathology and/or social outcomes

Identified studies reported associations between two domains of social cognition - emotion processing (social-perceptual part) and theory of mind (social-cognitive part) - and psychopathology and/or social outcomes. An overview of the reported links between social cognition and the domains of interest is presented in Table 2.

**Table 2:** Domains of social cognition in individuals with 22q11DS linked to psychopathology and social outcomes

Author (year)	N	Age M (SD), (range)	IQ (SD), (range)	Social Cognition	Social Cognition – Psychopathology			Social Cognition - Social Outcomes		
				Task	Instrument	Measure	Association	Instrument	Measure	Association
				**EP**						
Schneider et al. (2017) [56]	35	18.06 (3.40), (11–24)	(−)	BFRT BFRT	SPQ SPQ	paranoid schiz.symptoms negative schiz.symptoms	*r* = − 0.362, *p* = 0.022 *r* = −0.321, *p* = 0.060	DAS	severity of negative performance beliefs	*r* = −0.388, *p* = 0.021
Zaharia et al. (2018) [58]	75	T1 12.81 (3.57),(−)	71.96 (11.25),(−)	Face Discrimination Task:correct answers on CD trials	PANSS	negative symptoms	*r* = −0.293, *p* = 0.02	PANSS negative	used as measure of social abilities	*r* = − 0.293, *p* = 0.02
				Face Discrimination Task	A-CBCL	internalizing, anxious/depressive symptoms	0			
Vangkilde et al. (2016) [49]	29	15.7 (2.8), (−)	79.52 (−), (35–113)	ERT ERT	SIPS	negative prod.symptoms	d.r.	ABAS Social (p/s.r.) SRS Total (p.r.)	social functioning social skills	0 0
Badoud et al. (2017) [55]	28	17.79 (2.89), (11–21)	75.33 (11.66),(−)	Pictures of Facial Affect	SPQ	schizitypal traits	0	VABS (p.r.)	communication, daily living skills, socialization	0
Weinberger et al. (2018) [59]	44	20.7 (6.0), (12–33)	77.2 (11.0),(−)	Social Cognition Total: (Emotion Ident., Differention Age Differentiation)	SIPS SOPS	negative symptoms negative prod. symptoms (total negative score)	0 0	VABS (p.r.)	communication, daily living skills, socialization	n.r.
Campbell et al. (2011) [50]	50	10.99 (2.9), (6–17)	65.8 (9.3), (40–94)	Face Process Skills Battery	SDQ	emotional problems	n.r.	SDQ (p.r.)	peer relationship problems	*r* = 0.33, *p* < 0.03
Campbell et al. (2015) [54]	24	16.75 (3.14), (12–21)	75.9 (14.9), (56–115)	EAT	SDQ	emotional problems	n.r.	SDQ (p.r.)	peer relationship problems	beta = 0.69, *p* < 0.05
Dubourg et al. (2018) [57]	22	20.3 (5.2), (12–32)	74 (12),(−)	BFRT BFRT BFRT BFRT	DICA, SCID-I K-SADS-PL SIPS SIPS	psychiatric disorders psychiatric disorders positive prod. symptoms negative prod. symptoms	n.r. n.r. n.r. n.r.	SRS (p.r.)	social skills	0
Shashi et al. (2012) [53]	66	10.5 (2.6), (−)	(−)	DANVA	CBCL, C-DISC	psychiatric disorders	n.r.	SSRS Total (p.r.)	social skills	0
				**ToM**						
Vangkilde et al. (2016) [49]	29	15.7 (2.8), (−)	79.52 (−), (35–113)	TASIT Total TASIT Total	SIPS	negative prod. symptoms positive prod. symptoms	d.r. 0	ABAS Social (p./s.r) SRS Total (p.r.)	social functioning social skills	0 0
Badoud et al. (2017) [55]	28	17.79 (2.89), (11–21)	75.33 (11.66), (−)	The Director Task The Director Task	SPQ SPQ	negative schiz. traits disorganised schiz. traits	RT, *r* = 0.425, *p* = 0.062 RT, *r* = 0.393, *p* = 0.087	VABS (p.r.)	communication, daily living skills, socialization	0
(without participants on antipsychotic medication)				The Director Task The Director Task The Director Task	SPQ SPQ SPQ	positive schiz. traits negative schiz. traits disorganised schiz. traits	*r* = 0.464, *p* = 0.046 *r* = 0.598, *p* = 0.007 *r* = 0.576, *p* = 0.010			
Ho et al. (2012) [52](SUNY) (UCLA)	63			The Animations Task:						
	34 29	17.1 (1.9), (14–22) 13.7 (5.5), (6–25)	74.5 (13.2), (−) 80.5 (13.7), (−)	Intentionality Appropriatedness	SIPS SIPS	negative prod. symptoms positive prod. symptoms	0 0	SRS (p.r.) SRS (p.r.)	social skills social skills	*r* = −0.283, *p* < 0.05 *r* = −0.314, *p* < 0.05
Campbell et al. (2011) [50]	50	10.99 (2.9), (6–17)	65.8 (9.3), (40–94)	false-belief stories	SDQ	emotional problems	n.r.	SDQ (p.r.)	peer relationship problems	*r* = 0.3, *p* < 0.03
Campbell et al. (2015) [54]	24	16.75 (3.14), (12–21)	75.9 (14.9), (56–115)	PST (false-belief stories)	SDQ	emotional problems	n.r.	SDQ (p.r.)	peer relationship problems	0

*ABAS* The Adaptive Behavior Assessment System, *ACBCL/CBCL* Adult/child Behavior Checklist, *BFRT* Benton Face Recognition Test, *C-DISC* Diagnostic Interview Schedule for Children, *CD* configural different, *DANVA* Diagnostic Analysis of Non-Verbal Accuracy, *DAS* Dysfunctional Attitude Scale, *DICA* Diagnostic Interview for Children and Adolescents, *d.r.* descriptive report, *EAT* Emotion Attribution Task, *EP* emotion processing, *ERT* Emotion Recognition Task, *K-SADS-PL* Schedule for Affective Disorders and Schizophrenia for School Age Children, *n.r.* not reported, *PANSS* Positive and Negative Symptoms Scale, *p.r.* parent rated, *PST* Picture Sequencing Task, *RT* response time, *SCID* Structured Clinical Interview for DSM IV, Axis I Disorders, *SDQ* Strengths and Difficulties Questionnaire, *SIPS* Structural Interview for Prodromal Symptoms, *SPQ* Schizotypal Personality Questionnaire, *s.r.* self rated, *SRS* Social Responsiveness Scale, *SSRS* Social Skills Rating System, *TASIT* The Awareness of Social Inference Test, *ToM* Theory of Mind, *VABS* Vineland Adaptive Behavioral Scales, *0* no significant association (*p* > 0.05)

#### Emotion processing - social-perceptual (affective) part

In total, five papers reported associations between social-perceptual aspects of social cognition and *psychopathology*. Four of these studies found significant associations between face and emotion recognition and negative symptoms [49, 56, 58] and paranoid schizotypal symptoms [56]. No association was found between this aspect of social cognition (specifically, face discrimination) and internalizing, anxious/depressive traits [58].

In the domain of *social outcomes,* three papers reported significant associations with social cognition. Specifically, emotion processing was associated with peer relationship problems [50, 54] and with the severity of negative performance beliefs [56]. In contrast, four studies found no significant association between the social-perceptual aspect of social cognition and any social outcome (Table 2).

#### Theory of mind (ToM) - social-cognitive (reflective) part

Three papers reported specific associations between this aspect of social cognition and *psychopathology.* Associations were reported with the severity of negative symptoms [49, 55] as well as disorganised traits (odd behavior and speech) [55]. One study reported that upon removing participants who were on antipsychotic medication, previously marginal associations to positive, negative, and disorganisation schizotypal traits became significant [55]. No associations were found between this aspect of social cognition and attenuated positive symptoms of psychosis [49, 52].

Concerning social outcomes, reports are divided: two studies found associations to peer relationship problems [50, 52], while three papers found no associations to any of the social outcome measures [49, 54, 55]. Studies with confirmed links between the social-cognitive part of social cognition and social outcomes included larger samples (*n* > 50) (Table 2).

### Links between social cognitive domains and other variables

Six studies presented the impact of other variables (age, IQ, working memory (WM), executive function (EF)) on social cognition in individuals with 22q11DS (Table 3). Both aspects of social cognition were mainly associated with age. Zaharia et al. pointed that accuracy on configural different (CD) trials (comparing original face photo to the versions of face photos with eyes/mouth being moved up, down, further or closer) significantly increased with age only in typically developing participants compared to the affected individuals [58]. Most studies found no link to IQ. Emotion processing was related to WM and grammar reception [50]; ToM was associated with EF [54] and grammar reception [50] - each aspect of social cognition by a single study (Table 3). Assessments of neurocognition and social cognition are presented in the Additional file 1.

**Table 3:** Domains of social cognition in individuals with 22q11DS linked to other variables

Social Cognition						
Author	N	Age M (SD), (range)	IQ (SD), (range)	Task	Variable	Association
				**EP**		
Vangkilde et al. (2016) [49]	29	15.7 (2.8), (−)	79.52 (−), (35–113)	ERT	IQ	*r* = 0.43, *p* = 0.02
Zaharia et al. (2018) [58]	75	T1 12.81 (3.57), (−)	71.96 (11.25), (−)	Face Discrimination Task	IQ	0
Shashi et al. (2012) [53]	66	10.5 (2.6), (−)	(−)	DANVA	IQ	0
Badoud et al. (2017) [55]	29	17.79 (2.89), (11–21)	75.33 (11.66), (−)	Pictures of Facial Affect	IQ	0
Vangkilde et al. (2016) [49]	29	15.7 (2.8), (−)	79.52 (−), (35–113)	ERT	age	F = 10.58, *p* = 0.002
Campbell et al. (2011) [50]	50	10.99 (2.9), (6–17)	65.8 (9.3), (40–94)	Face Process Skills Battery		
				Identity	age	beta = 0.235, *p* < 0.005
				Gaze	age	beta = 0.226, *p* < 0.02
				Facial speech	age	beta = 0.242, *p* < 0.03
Zaharia et al. (2018) [58]	75	T1 12.81 (3.57), (−)	71.96 (11.25), (−)	Face Discrimination Task	age	d.r.
Badoud et al. (2017) [55]	29	17.79 (2.89), (11–21)	75.33 (11.66), (−)	Picturres of Facial Affect	age	0
Campbell et al. (2011) [50]	50	10.99 (2.9), (6–17)	65.8 (9.3), (40–94)	Face Process Skills Battery	WM	*r* = 0.44, *p* < 0.001
Campbell et al. (2011) [50]	50	10.99 (2.9), (6–17)	65.8 (9.3), (40–94)	Face Process Skills Battery	grammar reception	*r* = 0.646, *p* < 0.0005
Shashi et al. (2012) [53]	66	10.5 (2.6), (−)	(−)	DANVA	EF	0
Shashi et al. (2012) [53]	66	10.5 (2.6), (−)	(−)	DANVA	verbal learning and memory	0
Shashi et al. (2012) [53]	66	10.5 (2.6), (−)	(−)	DANVA	sustained attention	0
				**ToM**		
Ho et al. (2012) [52]	34	17.1 (1.9), (14–22)	74.5 (13.2), (−)	The Animations Task:		
				Intentionality (SUNY)	IQ	*r* = 0.511, *p* < 0.01
				Appropriatedness (SUNY)	IQ	*r* = 0.463, *p* < 0.01
				Appropriatedness (random)	IQ	*r* = 0.453, *p* < 0.01
Ho et al. (2012) [52]	29	13.7 (5.5), (6–25)	80.5 (13.7), (−)	The Animations Task:		
				Intentionality (UCLA)	IQ	0
				Appropriatedness (UCLA)	IQ	0
Vangkilde et al. (2016) [49]	29	15.7 (2.8), (−)	79.52 (−), (35–113)	TASIT	IQ	0
Badoud et al. (2017) [55]	29	17.79 (2.89), (11–21)	75.33 (11.66), (−)	The Director Task	IQ	0
Campbell et al. (2011) [50]	50	10.99 (2.9), (6–17)	65.8 (9.3), (40–94)	False-belief stories	age	*r* = 0.61, *p* < 0.0005
Vangkilde et al. (2016) [49]	29	15.7 (2.8), (−)	79.52 (−), (35–113)	TASIT	age	*r* = 0.35, *p* = 0.07
Ho et al. (2012) [52]	63			The Animations Task:		
(SUNY)	34	17.1 (1.9), (14–22)	74.5 (13.2), (−)	Intentionality (SUNY, UCLA)	age	0
(UCLA)	29	13.7 (5.5), (6–25)	80.5 (13.7), (−)	Appropriatedness (SUNY, UCLA)	age	0
Badoud et al. (2017) [55]	29	17.79 (2.89), (11–21)	75.33 (11.66), (−)	The Director Task	age	0
Campbell et al. (2015) [54]	24	16.75 (3.14), (12–21)	75.9 (14.9), (56–115)	PST (false-belief stories)	EF	beta = 0.44, *p* < 0.01
Campbell et al. (2011) [50]	50	10.99 (2.9), (6–17)	65.8 (9.3), (40–94)	False belief-stories	grammar reception	*r* = 0.347, *p* < 0.015

*DANVA* Diagnostic Analysis of Non-Verbal Accuracy, *d.r.* descriptive report, *EAT* Emotion Attribution Task, *EF* executive function, *EP* emotion processing, *ERT* Emotion Recognition Task, *FERT* Facial Emotion Recognition Test, *PST* Picture Sequencing Task, *TASIT* The Awareness of Social Inference Test, *ToM* theory of mind, *WM* working memory, *0* no significant association (*p* > 0.05)

## Discussion

We systematically reviewed studies that investigated the links between social cognitive domains and psychopathology and/or social outcomes in individuals with 22q11DS. Additionally, we included reported links between social cognition and other (neurocognitive) variables or age. Results can be summarized in the following points: 1) Ten identified studies involved assessments of three aspects of social cognition: emotion processing (social-perceptual (affective) part), ToM (social-cognitive (reflective) part), social perception and reported associations between two aspects of social cognition (emotion processing and ToM) and psychopathology and/or social outcomes. 2) With respect to psychopathology, associations were confirmed mainly with the severity of negative symptoms of psychosis. Concerning the relationship with positive symptoms of psychosis or emotional symptoms (anxiety/depression), the current state of research in the field of 22q11DS is limited and equivocal. 3) Results regarding the links between social cognitive domains and social outcomes are inconsistent. 4) Most studies point to an impact of age on social cognitive domains. Results will be discussed in the context of the existing body of knowledge on 22q11DS and schizophrenia research. Finally, clinical implications with preventive and treatment propositions will be considered.

### Social cognition in individuals with 22q11DS

Almost all included studies reported deficits in the assessed domains of social cognition. With respect to emotion processing (social-perceptual part), an exception involves a paper which investigated emotion processing using the paralanguage subtest (by the tone of voice) and found no significant difference to the control group [53]. This could imply that auditory emotion processing is intact in persons with 22q11DS. However, given that this was the only study that used auditory stimuli to assess this aspect of social cognition and reported that the sample size (*n* = 66) might not be large enough to detect significant group differences, this finding should be interpreted with caution and warrants further examination. In the domain of ToM (social-cognitive part), three studies reported no significant differences between individuals with 22q11DS and the control group [50, 54, 55] that were interpreted mainly in relation to the age of the sample, as it will be further discussed.

Regarding the assessments of social cognitive domains, two elements are notable. First, one paper compared 22q11DS individuals with/without autism spectrum disorder (ASD) to a control group using abstract, ambiguous visual stimuli to assess ToM abilities [52]. The finding confirms that individuals with 22q11DS, regardless of ASD, have significant ToM impairments in both the ability to explain purposeful behavior (Intentionality) and to accurately describe the events going on in the scene (Appropriateness). This was the only study that did not rely on verbal comprehension to assess ToM making it more optimal for children with the syndrome. Nevertheless, implicit mentalizing was also impaired in individuals with 22q11DS [52]. Second, social perception and attributional bias have been merely researched in individuals with 22q11DS: only one study assessed social perception [57] and no research examined attributional bias. Additionally, a paper that assessed social perception did not investigate the relation of this aspect of social cognition to psychopathology or social outcomes [57].

### Links between social cognitive domains and psychopathology

One of the main goals of our study was to review the association between social cognition and psychopathology, and three studies confirmed medium effect size associations between emotion processing and negative symptoms of psychosis [49, 56, 58]. This finding is also supported by the research of Jalbrzikowski et al. in which emotion processing was found to be specifically associated with negative symptoms [21]. A paper that did not observe a significant association between emotion processing and negative symptoms involved a total score for social cognition (including emotion identification, differentiation, and age differentiation) [59]. However, a significant association was observed between a global neurocognitive performance (GNP) score from the Penn Computerized Neurocognitive Battery (CNB) (including executive function, episodic memory, complex cognition, social cognition, and praxis speed) and negative symptoms. Specifically, GNP at baseline significantly predicted the emergence and persistence of negative symptoms at a fifteen-month follow-up [59]. Even if neurocognition and social cognition are conceptualized as distinct constructs, there is a significant overlap between them [19]. Altogether, these findings might imply that aspects of social cognition, specifically emotion processing, does play a role in the emergence of negative symptoms, however, in a manner that also involves other neurocognitive domains. Theory of mind was also found to be associated with negative symptoms [49, 55], which is in line with the reports from Frascarelli et al. [60] and the research on persons with schizophrenia [18]. Findings on risk and outcome in psychosis have already indicated that social cognitive domains might share the same etiological origin with negative symptoms [61], which seems to be in line with the current state of research on individuals with 22q11DS. Given that the severity of negative symptoms is a clinical characteristic of 22q11DS [62, 63], these findings highlight both aspects of social cognition (emotion processing and ToM) as possible targets for intervention strategies aimed at reducing the severity of negative symptoms in this population.

Two elements regarding the associations between social cognitive domains and positive or emotional symptoms should be noted. First, only one of the included studies reported a significant association between emotion processing and positive (paranoid) symptoms [56] and one reported a significant association only after removing participants who were on antipsychotic medication [55]. In contrast, two papers reported no significant association between either aspect of social cognition and positive symptoms [49, 52]. However, two studies that were not eligible for the present review (as they have not assessed social outcomes) reported links between emotion processing [64] and ToM [21] and positive symptoms in persons with 22q11DS. Research on schizophrenia grossly confirmed associations between social cognitive domains and positive symptoms, but these reported associations were also interpreted with caution warranting additional considerations [61, 65, 66]. By way of example, one study reported that high levels of positive symptoms were associated with the deficits in social cognition only in the presence of high levels of negative symptoms [67]. Second, only one included study investigated links between emotion processing and internalizing (anxiety/depression) symptoms in individuals with 22q11DS [58]. This paper reported no significant association among two domains, possibly due to the use of less thorough anxiety assessment [58]. Conversely, findings outside the scope of the present search reported links between unusual face processing (specifically, less time spent on eyes) and (self-reported) anxiety as well as (parent-reported) internalizing behavior [26, 27]. Additionally, the importance of emotional processes on the clinical expression of positive symptoms has been recognized for persons with 22q11DS [56] and in research on schizophrenia [68]. This implies that the relationship between social cognitive domains and positive symptoms is more complex and possibly requires consideration of other symptomatology. Given that the current state of research regarding the 22q11DS population is limited and given the predominant focus on positive and negative symptoms, future studies should consider focusing on an early tracking of emotional symptoms (e.g., anxiety/depression). For instance, a longitudinal design could elucidate the role of specific social cognitive deficits in relation to emotional symptoms at different time points and the possible path of progression to positive symptoms.

### Links between social cognitive domains and social outcomes

Our review highlights that the associations between social cognitive domains and social outcomes are inconsistent. Four studies found no significant association between emotion processing and social outcomes. In contrast, four papers confirmed the association [50, 54, 55, 58], but one article used the PANSS negative symptoms scores as a measure of social abilities [58], a tool initially designed to assess positive and negative symptoms of psychosis. A similar result was also found regarding the associations between ToM and social outcomes: three studies reported no significant association [49, 54, 55] to social outcomes and two papers observed significant links [50, 52]. This finding is not surprising considering the current state of research on schizophrenia in this respect. Although the prominent view is that social cognition has a mediating role between neurocognition and social functioning [32, 69, 70], several studies found no associations between these two domains [43, 47, 48]. Both findings invite for a closer understanding of the reported results. For example, social cognition (social perception) was specifically predictive of work, but not of social functioning in persons with psychosis [43]. Furthermore, the severity of symptoms reduced the strength of the association between social cognition and social outcomes [48]. Results of the present review are divided for any conclusive interpretation in this respect. However, the studies that did observe significant association between both parts of social cognition and social outcomes involved larger samples, and could therefore be accounted as more representative. It can be that the association between two respective domains changes at different time points and is influenced by the severity of symptoms, which warrants further investigation in population with 22q11DS..

It should be noted that given the highly complex nature of social outcomes (skills, competence, functioning), the operationalization of both constructs in an ecologically valid manner that can be compared across studies remains challenging [47]. In the present review, the social outcomes of individuals with 22q11DS were assessed by parent ratings in almost all included studies. At the same time, discrepancies regarding social outcomes in parent vs. teacher [71], or parent vs. child and sibling were observed in the group of participants with 22q11DS [72]. Therefore, it is questionable whether the parent-ratings of social outcomes are sensitive enough to observe a specific association with social cognition in individuals with 22q11DS. In this respect, suggestions regarding the need for multi-rater assessments, using multiple instruments for social-behavioral functioning have already been made [61] and should be considered in future studies.

### Links between social cognitive domains and other variables

In the terms of relationship to other tested variables (age, IQ, WM, EF), age emerged to be significantly associated with social cognition in most included studies. Older participants were found to perform better in emotion processing tasks [49, 50]. Additionally, a longitudinal analysis pointed that individuals with 22q11DS from the age of 6 years old improved to a lesser extent than typically developing controls in emotion (configural) processing (comparing original face photo of a woman to the versions of photos of the same woman, but with eyes/mouth being moved up, down, further or closer) [58]. With respect to ToM, several studies reported no significant group differences that was interpreted in relation to age. Specifically, Badoud et al. reported that both groups performed low on a perspective taking task [55] and Campbell et al. observed that both children with 22q11DS and controls passed simpler (first-order) false-belief tasks [50]. All the children who failed ToM tasks were in the age group between 6 and 9 years old. There is a need for additional longitudinal studies to better understand the developmental trajectories of social cognitive domains in persons with 22q11DS (but see [33]). Even though the current state of research is still modest for conclusions in this respect, reported findings point to social cognitive developmental deficits or delays that seem to manifest significant differences to typical development before the age of 6 years old in children with the syndrome. This implies that preventive strategies are to be considered very early and prior to the specified age.

Interestingly, only two included studies reported associations between emotion processing [49] and ToM [52] and IQ, and five papers found no association between these domains [49, 52, 53, 55, 58]. The article which observed a significant association between ToM and IQ reported different results at two sites depending on the format of the instruction provided to the individuals with 22q11DS [52]. Given that individuals with 22q11DS are in most cases characterized by borderline intellectual functioning and mild intellectual disability, this finding highlights that special consideration should be taken regarding the way in which ToM is assessed and the choice of instrument for this specific population. Concerning the association between neurocognition and social cognition, findings are limited as only three included papers investigated this association [50, 53, 54]. Links were reported between emotion processing and WM [50] and between ToM and EF [54]. It would be useful to further understand the influence of neurocognition on social cognitive domains using longitudinal designs.

### Clinical implications and treatment suggestions

The current state of research on interventions for persons who are at the risk for schizophrenia/psychosis recommends approaches that can be applied early in life in order to improve concrete aspects of the atypical developmental trajectory, and prior to the appearance of severe psychopathology, functional impairment, or diagnosable disorder [73]. Given that individuals with 22q11DS are at increased risk for the development of psychosis, early detection of vulnerable domains, such as social cognition, and the impact of other variables during the course of development would possibly lead to the timely implementation of strategies that would improve concrete aspects of development. Additionally, it has been reported that parents of children with 22q11DS can be at increased risk for burnout [74]. Considering the role of caregivers in the development of the mentalizing capacity of children [75], which might be compromised in the case of overwork or burnout, intervention strategies may consider involving the affected children and their parents.

Although associations among studied domains in a population with 22q11DS are complex and sometimes inconsistent, they can be informative of possible preventive strategies. Concerning social cognition specifically, a proposition has already been made for the inclusion of the assessment of this domain as a potential biomarker and a screening tool for various clinical conditions in the general population [23]. In the context of individuals with 22q11DS, a longitudinal monitoring of this domain in affected children could be used to broaden our understanding of clinical and social outcomes.

With respect to the concrete preventive strategies, research on the effectiveness of specific interventions in this specific population is still modest. Therefore, possible strategies can currently be made mainly in the form of psychoeducational recommendations. For example, studies in the present review confirm associations between both social cognitive domains and predominantly negative symptoms of psychosis [49, 55, 56, 58]. Even if additional (especially longitudinal) research is required, the current body of evidence suggests that early intervention focusing on emotion perception and ToM could possibly lead to the prevention of negative symptoms, which in turn may impact better functional outcomes. Interventions targeting emotion processing designed specifically for children with 22q11DS, such as vis-à-vis program [76] have already been proposed. Additionally, social cognitive remediation appropriated for children has also been suggested [77]. In the field of schizophrenia, a combined intervention for neurocognition and social cognition has been proposed [78, 79], notably including computer-aided sessions or individual/group settings involving discussions on social scenes, interactions, or role-playing.

At the same time, worthy of note is that early deficits related to social cognitive domains are often coupled with the challenges of various physical conditions that are characteristic of the syndrome and that affect both children and their parents. For this reason, a possible strategy could consider engaging immediate surrounding in a manner that would create (an early) preventive environment. With respect to the domains of social cognition specifically, parental embodied mentalizing (PEM) [80] and mentalization based treatment (MBT) [75] can be of relevance. Both approaches emphasize the interpersonal environment for emotion regulation and the development of mentalizing capacity. Additionally, MBT has been proven to be effective for individuals with psychosis especially during the early phase of illness [81]. However, heterogeneity of the 22q11DS population and impaired cognitive capacities of some individuals should be underlined and would require further investigation in terms of preventive strategies. Equally, given that aspects of social cognition (e.g., ToM) continue to improve during adolescence and early adulthood [31], but at the same time may manifest developmental delays already in childhood in the 22q11DS population, age is an additional factor to be considered. This may also mean that interventions, entailing specific (neurocognitive and social cognitive) domains for the specific age, could be personalized for each child (family) affected with the syndrome. The importance of including the environment (parents and other clinicians) is not only essential for the necessary improvements of early social cognitive deficits, but also for the timely detection of changes in the behavior that might lead to symptoms. Rather than focusing primarily on the psychiatric profiles and diagnosis, early uncovering and understanding of such behaviors is important for implementing preventive and intervention strategies, as it has already been recognized in the 22q11DS population [7].

### Strengths, limitations, future orientation

The present review points to several strengths and limitations regarding the current state of research on social cognitive domains in individuals with 22q11DS and its links with psychopathology and social outcomes. With respect to participants, affected groups were recruited mainly from parent associations (not mental-health related) and control groups from the local community using the appropriate selection strategy, therefore minimizing the risk of selection bias. All included studies involved individuals with confirmed 22q11DS, including age- and gender-matched controls, which eliminated confounding in this respect. Most studies did not match the groups on IQ and did not control for this variable in group comparisons. Given that difference in IQ is an inherent property of the grouping, it would be statistically improper to control for differences in this aspect in the comparisons [82]. Studies in the review focused on two aspects of social cognition - emotion processing and theory of mind - using tasks similar to those used in schizophrenia research. This makes it relatively easy to contrast the obtained findings with those acquired on individuals with schizophrenia/psychosis.

At the same time, investigation of the links between chosen domains is complex and several points need to be highlighted. First, except for two studies that employed longitudinal designs [58, 59], all included papers used a cross-sectional design that intrinsically provides limited quality of evidence. Due to the heterogeneity of included studies and reported data, it was not possible to conduct a meta-analysis, thus the review is a narrative synthesis of provided evidence. Clinical diversity of the studied population is a major issue that has likely contributed to the inconsistency of the reported results. Factors such as psychiatric comorbidities, the severity of symptoms, use of medication could be considered as potential confounders. For example, studies varied on the inclusion of participants with a psychotic disorder or whether they reported other psychiatric comorbidities and use of medication. Additionally, none of the mentioned factors could be suggested as a potential moderator due to underreporting and the lack of precisely conducted moderator analysis. In order to avoid these limitations, future research could consider classifying subgroups of affected individuals based on their clinical features and/or social outcomes. This would possibly clarify the nature of the reported associations across groups of affected individuals, particularly in the context of formerly suggested longitudinal designs.

Second, overlapping and distinct aspects of the concepts behind chosen domains including diverse sensitivity and precision of the instruments used for evaluation is a recurrent theme in the field. This invites special consideration that is beyond the focus of the present study and requires separate research. In most included studies, domains of interest were assessed by validated instruments specifically designed for the assessment of each respective domain. The exception involves a study that used the PANSS negative symptoms scale [12], which is designed for the assessment of negative symptoms of psychosis as the measurement of social outcomes [58]. Another paper focusing on dysfunctional beliefs was included and categorized under the social outcomes measurement [56]. However, included studies varied in the precision of the instruments that were used. Given the wide range and complexity of the chosen constructs, future studies should employ strategies that minimize the overlapping aspects of these domains and try to focus the investigation on the facets that exclusively stand for each construct. By way of example, excluding decreased experience of emotions and social withdrawal subscales from the measurements of psychopathology, as these may rather reflect social cognition and social outcomes respectively. Similarly, in instruments that are used for the assessments of both psychopathology and social outcomes (e.g., SDQ), it should be useful to divide analysis for each subscales and items that uniquely stand for each domain.

Third, there is a clear gap in knowledge on social perception and attributional bias for this specific population, which should be addressed in future research. Concerning the specific tools for the assessment of social cognition, only two studies reported using instruments specifically designed for research with children with developmental disorders [50, 54]. Limitation regarding the choice of stimuli, tools, and tasks that are used for social cognitive domains has already been acknowledged in the field. Regarding emotion processing, one of the concerns is that static portraits of face photos do not match the complexity of variations in facial expressions in daily life or social interactions. Regarding the assessment of ToM, a major criticism is that participants have an observer role and are required to infer the mental states of individuals with whom they are not interacting. Additionally, certain tasks used for ToM rely on the verbal understanding, other neurocognitive measures (e.g., WM), or are influenced by the format of directions provided to the participants (due to their IQ range), which should be considered in the research for this specific population. The use of dynamic stimuli and more naturalistic assessments (e.g., clinical interviews to evaluate mentalization) could be an option to minimize these limitations. The proposition of the SCOPE study [24] for measures with the strongest psychometric properties [66, 83, 84] could also be of relevance. At the same time, recommendation for the most appropriate instruments for social cognitive domains specifically for the 22q11DS population would be useful in order to overcome these limitations.

Fourth, concerning the investigated links in the present study, six included papers reported on the links between social cognitive domains and psychopathology [49, 52, 55, 56, 58, 59] and all but one paper [59] reported associations with social outcomes. The inclusion criteria of the present review required studies to involve assessments of all domains of interest and to report the links between social cognitive domains and psychopathology and/or social outcomes. In the selection process, 12 studies were excluded as they have not investigated nor reported the associations of interest (inclusion criterion number four), even though they had assessed all domains of interest. In contrast, several studies that were excluded due to the lack of the assessments of either psychopathology or social outcomes, sometimes did report associations we were interested in. This was mainly regarding the links between social cognition and psychopathology. The inclusion of such papers would have broadened the scope of the review in a manner that warrants a new study. We tried to overcome this limitation by complementing discussion with the very findings from the excluded papers and are confident that no data we were interested in was lost due to this limitation.

Finally, the cross-sectional design used by most included studies does not allow a possibility for causal interpretations. Future longitudinal research should be conducted in order to have the possibility to investigate the directionality of the observed findings. This will provide a better understanding of the nature behind reported links and will secure timely preventive strategies for this population. Specifically, the acquired knowledge will inform on the necessity of the concrete prevention strategy targeting a specific domain for the specific age and will warrant the evolution of preventive strategies for individuals with 22q11DS.

## Conclusion

Research on the links between social cognitive domains and psychopathology as well as social outcomes in persons with 22q11DS is in its developing stage since only ten studies fitted the eligibility criteria for the present review. Interestingly, even when studies involved domains of interest, many did not investigate the associations to social cognition specifically, but focused on the associations between other aspects (i.e., psychopathology and social outcomes, neurocognitive variables and social outcomes). Along with the current state of research on schizophrenia, results of the present review provide sufficient evidence that social cognition is domain of interest to broaden our understanding of clinical outcomes and social difficulties in persons with 22q11DS. Indeed, significant associations between both social cognitive domains (emotion processing and ToM) and negative symptoms have been reported by several studies [21, 49, 55, 56, 58, 60]. Concerning positive symptoms, the association seems to be more complex in that it possibly involves the impact of other aspects (e.g., neurocognition, emotional symptoms) and requires further research. Even though most studies focused primarily on investigating the associations between social cognitive domains and positive and negative symptoms of psychosis, the role of other symptoms and variables should not be underestimated. At the same time, due to the wide range and complexity of these respective domains, assessment strategies should aim to investigate the facets that uniquely represent each construct and minimize the potential overlap between them. Given that the affected persons are at risk for psychopathological manifestations and social difficulties, longitudinal studies that can monitor the development of social cognitive domains, the impact of neurocognition and emotional symptoms at different time points would provide a better understanding of affected persons and the progression to various clinical outcomes. Accumulated knowledge would provide informed decisions about timely preventive strategies that can be applied for individuals with 22q11DS and their environment.

## Supplementary Information

**Additional file 1.** Assessments of social cognitive domains, psyschopathology, social outcomes, and neurocognition [12, 13, 24, 30, 31, 66, 83–121].

## Data Availability

The datasets used and/or analyzed during the current study are available from the corresponding author on reasonable request.

## Abbreviations

22q11DS: 22q11 deletion syndrome
ABAS-II: The Adaptive Behavior Assessment System—Sec. Ed.
ABCL: Adult behavior checklist
ADHD: Attention deficit hyperactivity disorder
ADI-R: Autism Diagnostic Interview-Revised
ADOS: Autism Diagnostic Observation Schedule
ASD: Autism spectrum disorders
ASR: Adult self-report
BFRT: Benton Facial Recognition Test
BLERT: Bell Lysaker Emotion Recognition Task
CBCL: Child behavioral checklist
CD trial: Configural different trial
C-DISC: Diagnostic Interview Schedule for Children
DANVA: Diagnostic Analysis of Non-Verbal Accuracy
DAS: Dysfunctional Attitude Scale-Form A
DICA: Diagnostic Interview for Children and Adolescents
EAT: Emotion Attribution Task
EF: Executive function
EP: Emotion processing
ERT: Emotion Recognition Task
FD trial: Featural different trial
FISH: Fluorescence in Situ Hybridization
GAF: Global Assessment of Function
IAPS: International Affective Picture System
IQ: Intelligence quotient
K-SADS-PL: Schedule for Affective Disorders and Schizophrenia for School-Age Children - Present and Lifetime
PANSS: Positive and Negative Syndrome Scale
PRISMA: Preferred Reporting Items for Systematic Reviews and Metanalysis
PST: Picture Sequencing Task
QF-PCR: Quantitative Fluorescence Polymerase Chain Reaction
RT: Response time
SADS: Schedule for Affective Disorders and Schizophrenia for School-Age Children
SCID: Structured Clinical Interview for DSM IV, Axis I Disorders
SCOPE: Social Cognition Psychometric Evaluation
SDQ: Strengths and Difficulties, Questionnaire
SIPS: Structured Interview for Prodromal Syndromes
SOPS: Scale of Prodromal Symptoms
SPQ: Schizotypal Personality Questionnaire
SRS: Social Responsiveness Scale
SSRS: Social Skills Rating System
TASIT: The Awareness of Social Inference Test
ToM: Theory of Mind
VABS: Vineland Adaptive Behavior Scales
WM: Working memory
YSR: Youth self-report
