# Supplementary material for: Social cognition in individuals with 22q11.2 deletion syndrome and its link with psychopathology and social outcomes: a review
Source: BMC Psychiatry. 2021 Mar 6;21:130. doi: 10.1186/s12888-020-02975-5 (PMC7936464; doi:10.1186/s12888-020-02975-5)
Supplement: Supplementary file 1 — Additional file 1. Assessments of social cognitive domains, psyschopathology, social outcomes, and neurocognition [12, 13, 24, 30, 31, 66, 83–121]. [file 12888_2020_2975_MOESM1_ESM.pdf]

Additional file 1

Assessments of social cognitive domains

Assessments of emotion processing (EP) - social-perceptual (affective) part

Face Recognition - assessment of face discrimination ability of the participants. Tests used:

Benton Facial Recognition Test (BFRT) [85] - matching non-emotional unfamiliar faces in which the target face is compared to either identical or to photos of the target face taken from different angles or lighting conditions.

Face Discrimination (Jane) Task [86] - compares original face (of a woman called ´Jane´) with photos (of Jane) that have been manipulated in configural or featural manner. Possible trials involve: Configural Different (CD), Featural Different (FD), or Identical trials.

- CD trials - original Jane photo is compared to four new versions of Jane that have been modified in terms of the spacing between the facial features; the new versions portray one of the four following changes: the eyes or the mouth are moved either up or down; the eyes are either closer together or further apart.
- FD trials - original Jane photo is compared to four new portraits in which the eyes and mouth have been replaced with those from other persons.

Emotion identification - tests used or recommended:

Emotion Recognition Task (ERT) [87], Emotion Attribution Task (EAT) (see [88]), Pictures of Facial Affect [89], The Penn Emotion Recognition Test (ER-40) [84] - participants are presented with images of facial expressions depicting 'neutral' and several of six emotions (happy, sad, angry, fearful, surprise, disgust, shock). Participants are evaluated in their ability to identify the emotion (emotion recognition), to differentiate between different levels of emotion intensity (emotion differentiation) and/or to name it.

Bell Lysaker Emotion Recognition Task (BLERT) [83] - a tool recommended by the SCOPE study [24] due to the strong psychometric properties for emotion processing. In this test, participants are presented with twenty-one video-clips, each in the duration of ten seconds which present seven emotional states: happiness, sadness, fear, disgust, surprise, anger, or no emotion. Participants are assessed in their ability to correctly identify presented emotional states.

DANVA (paralanguage subtest) [90, 91] - assessment of emotion recognition by the tone of voice. In this assessment, a 10-year-old girl repeats the sentence: 'I am going out of the room now, but I will be back later,' in a manner that conveys one of four basic emotions.

MRC Face Processing Skills Battery [92] - examines four different aspects of face processing: Identity, Emotion, Eye gaze, and Facial Speech. The main analysis for this aspect of social cognition involved the number of correct answers (accuracy) and/or the response times. This tool is reported to be effective for research with children with developmental disorders.

Assessments of theory of mind (ToM) - social-cognitive (reflective) part

False-belief stories:

- The Sally-Anne [93] - first-order false-belief, examining beliefs different from one's own and the ability of a person to predict another's behavior.
- The Smarties task [93] - first-order false-belief, guessing what is in a box.
- The Chocolate task [93] - second-order false-belief, ability to think what a person falsely believes another person believes; only if previous two are passed.
- The Strange Stories task [93] - ability to interpret non-literal statements (false-belief, double-bluff, manipulation).

These false-belief tasks are reported to have good validity and fair to moderate reliability specifically for children with varying intellectual abilities and/or for those with developmental disorders.

Picture Sequencing Task (PST) (see [88]) - false-story/mentalizing is compared to three control story types (social-script, mechanical, and capture). Mentalizing is assessed by the ability of a person to understand that character's actions in a story are due to misinformation about a situation.

Control stories involve:

social-script - controlling for the ability to logically sequence social behavior.

mechanical stories - controlling for the ability to understand physical cause-and-effect relationships.

capture stories - controlling for the ability to inhibit misleading information.

The Hinting Task [66] - ten brief stories involving two characters. At the end of each story, one of the characters provides (a fairly clear) hint about the intention of the protagonists. Participants are asked what the protagonist in the story really wanted to say (with the hint s/he made). If the participant responds correctly, two points are given; if not, additional information is added to make the hint clearer. In the case of a correct response with an additional hint, one point is given; otherwise, an incorrect response amounts to zero points. The total score ranges from 0-20. The test was created for evaluating theory of mind (ToM) specifically for persons with schizophrenia. Given that the evaluator can read to the participants as many times as

needed to ensure a correct understanding, this test reduces the inference of possible memory impairment or verbal comprehension of participants.

Video-clips - depicting various social scenarios.

The Awareness of Social Inference Test (TASIT) [94] - professional actors perform everyday interactions that involve sincere, simple, and paradoxical sarcasm.
- Sincere clips convey congruence between what is literally said and the accompanying paralinguistic and facial cues.
- Simple and paradoxical sarcasm portray incongruence between what is said and accompanying paralinguistic and facial cues; simple and paradoxical sarcasm require recognizing and correctly interpreting cues such as facial expressions, tone of voice, gesture et cetera.

The Director Task [31] - participants are asked to select the right object that is visible to the director (according to his perspective) and to ignore the object invisible to him (even if it fits the instruction best). Main analyses involve accuracy (ACC) and response times (RT) for experimental trials.

The Animation Task [30] - four ToM and four random video-clips are shown in randomized order. Each video-clip consists of two triangles moving around on a white screen.
- ToM clip - triangles perform a scripted interaction of coaxing, seducing, mocking, or surprising. These scripts involve complex mental states that are not directly observable and involve interactions on a mental level.
- Random condition clips - triangles are moving without any purpose or interaction as: billiards, stars, tennis, or drifting.

This test does not use any verbal cues; assessment of implicit mentalizing.

Assessment of social perception

Processing of intact images from the International Affective Picture System (IAPS) is compared to scrambled IAPS images [95] (created using the Photoshop plugin).
- Participants are presented with both types of images and are asked to indicate whether the image is intact or scrambled using the MRI-compatible response box.
- Target images are divided into four categories (two based on content): social/non-social and (two based on valence): positive/ negative. Social images contained at least two human beings and non-social images did not include any humans.

Assessments of psychopathology

Strengths and Difficulties Questionnaire (SDQ) (emotional problems) [96]
Autism Diagnostic Observation Schedule (ADOS) [97]
Autism Diagnostic Interview-Revised (ADI-R) [98]
Structured Clinical Interview for DSM IV, Axis I Disorders (SCID-I) [99]
Child behavioral checklist (CBCL) [100]
Diagnostic Interview Schedule for Children (C-DISC) [101]
Global Assessment of Function (GAF) [102]
Schedule for Affective Disorders and Schizophrenia for School Age Children – Present and Lifetime (K-SADS) [103]
Structured Interview for Prodromal Syndromes (SIPS) [104]
Schizotypal Personality Questionnaire (SPQ) [13]
Youth/Adult self-report [105, 106]
Diagnostic Interview for Children and Adolescents (DICA) [107]
Adult behavioral checklist (ABCL) [106]
Positive and Negative Syndrome Scale (PANSS) [12]
Scale of Prodromal Symptoms (SOPS) [104]

Assessments of social outcomes

Strengths and Difficulties Questionnaire (SDQ) (peer relationship problems) [96]
Social Responsiveness Scale (SRS) [108]
Social Skills Rating System (SSRS) [109]
The Adaptive Behavior Assessment System—Sec. Ed. (ABAS-II) [110]
Vineland Adaptive Behavior Scales [111]
Dysfunctional Attitude Scale-Form A (DAS) [112]
Positive and Negative Syndrome Scale (PANSS) [12] (poor social abilities – negative symptoms subscale)

Assessments of neurocognition

Intellectual functioning (IQ):

Reynolds Intellectual Screening Test (RIST) [113]
Wechsler Intelligence Scale for Children 3$^{rd\ and\ 4th}$ edition (WISC-III, IV) [114, 115]

Working memory (WM):

Measured by performance on the digit span (from Wechsler Intelligence Scale for Children) [114, 115]

Grammar reception:

The Test for Reception of Grammar (TROG) [116] - measures understanding of grammatical contrasts. The tests are designed to remove contextual cues; therefore, a child is assessed only by grammatical structure in interpreting the sentence correctly. Each sentence presented to a child has four pictorial options; the items contain non- and -grammatical distractors that assist in determining if a child has a specific problem with grammatical understanding or low performance is due to other factors (i.e., poor attention or memory).

Executive function (EF):

Computerized planning task based on the Tower of London Test (ToL) [117] - participants are presented with a start configuration of three pegs of various heights and three balls of different colors. They are then instructed to rearrange the balls into a goal configuration using the touch screen. The start and the goal configurations appear simultaneously in the upper and lower half of the screen. The balls can only be moved one at a time and if there was no other ball on top. There is no time limit for the completion of the task.

NIMH-MATRICS battery [118] - used for the assessment of neurocognition in schizophrenia:

Executive function (EF):

Wisconsin Card Sorting Test (WCST) [119]

Verbal learning and memory:

California Verbal Learning Test- Children's Version (CVLT) [120]

Sustained Attention:

The Continuous Performance Test, identical pairs version (CPT-IP) [121]
